# RETRACTION: Estrogenic Effect of the Extract of QingYan Formula on Reproductive Tissues in Immature Mice

**DOI:** 10.1155/ecam/9831060

**Published:** 2025-12-03

**Authors:** Evidence-Based Complementary and Alternative Medicine

RETRACTION: Y. Zhao, H.-X. Zheng, Y. Xu, N. Lin, “Estrogenic Effect of the Extract of QingYan Formula on Reproductive Tissues in Immature Mice,” *Evidence-Based Complementary and Alternative Medicine*, 2019, https://doi.org/10.1155/2019/5493714.

The above article, published online on 8 January 2019 in Wiley Online Library (https://wileyonlinelibrary.com) has been retracted by agreement between the Editorial Board and John Wiley & Sons Ltd. The retraction has been agreed following an investigation into figure concerns initially raised on PubPeer [1]. Specifically,

• Figure 4(b) appears to contain unexpectedly repeated elements throughout all panels.
• Figure 5(b) appears to contain unexpectedly repeated elements in the ER*β* I and ER*α* III panels.

The authors were contacted and provided corrected images for Figures 4 and 5. However, the response did not satisfy our concerns and it is therefore retracted.

## References

[1] https://pubpeer.com/publications/10ED49F0CBF748F95FAA202DB4BFCB.

